# Assessing the feasibility of a community health worker-delivered mental health intervention for Latina immigrant women

**DOI:** 10.3389/fpubh.2025.1545922

**Published:** 2025-04-15

**Authors:** S. Adriana Pérez-Solorio, Juan Gudino, Georgina Perez, Serena Maurer, Barbara Baquero, Deepa Rao, India J. Ornelas

**Affiliations:** ^1^Department of Health Systems and Population Health, University of Washington, Seattle, WA, United States; ^2^Department of Global Health, University of Washington, Seattle, WA, United States

**Keywords:** mental health, Latina immigrants, community health workers, feasibility study, fidelity (performance)

## Abstract

**Introduction:**

Latina immigrants are at high risk for mental health concerns and also face barriers to accessing mental health resources. Community health workers are increasingly being used to provide culturally relevant mental health care in areas with a limited workforce.

**Methods:**

This study tested an implementation strategy of training and support community health workers (CHW) to deliver Amigas Latinas Motivando el Alma (ALMA), a community-based mental health intervention to reduce depression and anxiety among Latina immigrants. We trained five CHWs to deliver the ALMA intervention at two community-based organizations in central Washington. These CHWs then offered the program to Latina immigrants (*n* = 37) at each of their organizations with support from our bilingual research team. We collected survey data on CHW and participant characteristics, intervention fidelity, participant attendance, satisfaction, and changes in depression (PHQ-8) and anxiety (GAD-7) pre-and post-intervention.

**Results:**

Overall, training and supporting the CHWs to deliver the intervention was feasible. CHW completed all training sessions and delivered almost all of the program activities in the ALMA sessions. We found high participant engagement and satisfaction with the program. Mean depression scores (PHQ-8) decreased from 7.2 (mild severity) pre-intervention to 4.5 (minimal severity) post-intervention, and anxiety scores (GAD-7) decreased from 6.7 (mild severity) to 3.8 (minimal severity).

**Discussion:**

These results indicate that training CHW may be an effective way to broaden the reach and sustainability of the program. Future research should evaluate this approach in a larger trial with a more rigorous study design.

## Introduction

Latinas are at high risk for poor mental health and for those who are immigrants, rates of depression and anxiety only increase with time in the United States (1, 2). This is in part due to immigration-related stressors, including economic strain, family separation, social isolation, trauma exposure, and discrimination (3, 4). Latina immigrants also face significant barriers to accessing mental health care. Restrictive immigration policies and discrimination impact their ability to access to health care (5). Even when insured, there are a limited number of linguistically and culturally appropriate mental health care providers (6–8). Therefore, there is a need for linguistically and culturally appropriate mental health interventions for Latina immigrants that can be delivered by trusted providers in community settings.

ALMA is an intervention designed to prevent and reduce depression and anxiety among Latina immigrants by increasing social support and the use of evidence-based coping strategies. ALMA was developed in partnership with community-based organizations serving Latino immigrants over several years (9–11). The program was originally designed to be implemented by trained mental health and mindfulness professionals with at least master’s level training. The in-person group intervention was delivered over eight weekly sessions within trusted Latino-serving community-based organizations (CBOs). Another version of the program was delivered online over six weekly sessions. The curriculum is interactive and integrates aspects of Latino culture to foster participants’ sense of connection with family, home, culture, and fellow participants. Cultural elements include singing, art activities, sharing migration stories, family aphorisms (*dichos*), foods, and traditions. Sessions offer instruction and practice in evidence-based mindfulness and self-compassion practices focused on physical sensations, the breath, emotions, self-talk, eating, and movement, which have been proposed to be especially well-suited for the contextual factors that Latinas face (e.g., acculturative stress, traumatic stress). All sessions are designed to promote social connection by encouraging participants to share experiences and knowledge with one another in large and small group settings, discuss emotional challenges in their lives, and recognize commonalities across different experiences. ALMA was tested in a NIH-funded delayed intervention control trial in collaboration with two CBOs and results showed that ALMA had a significant impact on reducing depression symptoms and preventing the increase of anxiety symptoms (12). The intervention was delivered with high fidelity, and participants reported high levels of satisfaction and increased use of coping strategies in both quantitative and qualitative data (13, 14).

Since the trial, CBOs have expressed interest in offering the ALMA program to Latina immigrants in their communities. However, there is a limited workforce of master’s level, bilingual facilitators with mental health and mindfulness expertise (15, 16). Therefore, interested organizations face significant barriers to adopting ALMA as it was originally implemented. Furthermore, few previous studies have assessed the feasibility of using community health workers (CHW) to deliver community-based mental health interventions. In this study, we assessed the feasibility of a new implementation strategy for the ALMA program: training and supporting CHWs embedded in CBOs to deliver the program. CHWs, defined as lay health professionals that share similar demographic characteristics to the communities they serve, are well equipped to increase social support and reduce mental health disparities in underserved communities due to their ability to build trust, connect culturally with community members and identify local needs (16, 17). CHWs are also known to be a source of social support, and have strengths in providing culturally appropriate care education and navigating complex health and social systems (17, 18). In this pilot study, we assessed the feasibility of training and supporting CHW as an implementation strategy to deliver ALMA by assessing participant and implementation outcomes.

## Methods

### Preparation for the pilot study

Two CBOs in Central Washington expressed interest in having their staff trained to deliver the ALMA intervention to their clients given the urgent need for mental health support in their communities. Both CBOs are trusted providers of various educational programs, workshops, and referral services for Latino immigrants in their communities. In preparation for the pilot study, Latina immigrants who were staff at both CBOs were offered the six session online version of ALMA so they could assess whether the program would be a good fit for their clients. In feedback sessions, these CBO staff members expressed high satisfaction with the intervention and a strong desire to deliver the program in-person with the communities their organizations serve. With their input, our team developed a facilitator training program for CHWs at both organizations. Each CBO collectively chose CHWs from their organizations to participate in the training based on their previous experience with the ALMA curriculum and related programming for Latina immigrant women. One organization chose 2 CHWs to be trained and the other organization chose 3 CHWs to be trained. All but one of the CHWs chosen had been a participant in the ALMA program at their organization.

### Training community health workers to deliver ALMA

Our team offered these CHWs a facilitator training, with online and in-person components, to support them in offering ALMA to their communities. We provided CHW participants from each CBO with a training manual which included detailed scripts and instructions for each of the six in-person ALMA sessions, implementation recommendations, and lists of session materials. We also purchased and prepared intervention materials for the organizations. The online training was delivered by previous ALMA facilitators (mindfulness and mental health experts) in seven weekly 2-h sessions. This mirrored the ALMA intervention sessions which were also delivered once per week in 2-h sessions. The first session offered an introduction to the ALMA program and to facilitation principles (e.g., regarding practicing self-awareness and self-care, relying on one another for support, and cultivating connection in each ALMA session). In subsequent sessions, they reviewed each ALMA session and practiced facilitating the activities in that session. Following the seven online sessions, we offered a two-day in-person training “retreat” during which the CHWs had an opportunity to practice, discuss, and lead ALMA activities and practices with one another. Facilitators were able to observe CHWs leading sessions during this training but did not formally assess their level of competency.

### Supporting community health workers to deliver ALMA

After completion of the training, the facilitator teams from each CBO offered a six session in-person version ALMA to Latina immigrants involved with their organizations and wider communities. During this time, research team members met with each organization’s CHW facilitators weekly to offer guidance, suggestions and support. These meetings generally occurred 3–5 days after each ALMA session and provided an opportunity for CHWs to debrief the previous session and ask questions in preparation for the upcoming session.

### Participant recruitment and data collection

Participants in the ALMA intervention offered by the CHWs were recruited from their two community-based organizations serving Latino immigrants in central Washington. Each community organization was asked to recruit at least 20 participants. Community organization staff recruited participants from among their organization’s clients, such as those who participated in other education programs, and through word-of-mouth. Participants were screened for eligibility criteria, which included identifying as a Latina, being a Spanish speaker, and being 18 years of age or older. Eligible participants were asked to provide consent to participate in the study.

### Survey procedures and materials

Participants in the ALMA intervention were asked to complete a baseline survey which included questions on demographics, depression, anxiety, mindfulness-based coping strategies, and social support. In the month following the intervention, participants were contacted again to complete a post-intervention survey. The post-intervention survey included additional questions about participant satisfaction and skills learned in the ALMA sessions. Surveys were conducted by telephone by a Spanish-speaking Latina research team member who was not involved in intervention training or delivery. Participants received a $50 gift card for each survey they completed. Community Health Workers also completed a brief survey with questions on their demographic and work characteristics.

### Implementation outcomes

#### Community health worker characteristics

Facilitators were asked demographic information, including country of birth, years in the U.S., age, languages spoken and length of time working at the CBO. We also tracked their attendance at training and intervention sessions.

#### Fidelity

We assessed intervention fidelity through our weekly CHW facilitator support meetings and a weekly survey completed by the CHW facilitators after each ALMA session. The survey included questions about participant attendance and open-ended fidelity questions inquiring about anything that occurred during each session that may have deviated from typical intervention procedures (e.g., *was there something unusual or unexpected with the session*, and *were you able to complete all the session components*). Upon completion of the first ALMA session each CHW team facilitated, members of our research team guided the CHW facilitators through the fidelity survey to model the type of information that should be included. After that, the CHW teams completed the surveys on their own and were offered the opportunity for further discussion of the survey questions during our weekly CHW facilitator support meetings. We chose not to record or observe the sessions in order to protect participant privacy.

### Participant outcomes

#### Participant characteristics

Participants provided demographic information, including country of birth, years in the U.S., age, education, employment status, previous month’s household gross income and whether there was a partner living in the home.

#### Participant satisfaction

Participants in the ALMA intervention were asked 12 five-point Likert-scale questions about their experience in the program, including logistics, perceived efficacy of the program, and overall enjoyment of the program.

#### Skills learned

Participants in the ALMA intervention were asked 9 questions about how frequently they utilized the skills taught during ALMA. Response options used a 5-point Likert-scale ranging from never to almost every day.

#### Depressive symptoms

Depressive symptom severity was measured with the 8-item Patient Health Questionnaire-8 (PHQ-8), which asks how frequently participants experienced common symptoms of depression in the past 2 weeks. Response options range from never (0) to almost every day (3). Total scores of 10 and over are categorized as moderate to severe. This measure has been evaluated among racially and ethnically diverse populations, including Latinos, and found to effectively detect and monitor depression (19).

#### Anxiety symptoms

Anxiety symptom severity was measured with the 7-item Generalized Anxiety Disorder-7 scale (GAD-7) which assesses the frequency of common anxiety-related symptoms over the past 2 weeks. Response options range from never (0) to almost every day (3). Total scores of 10 and over are categorized as moderate to severe. The GAD-7 has been found to have good reliability and validity among Spanish-speaking Latinos (17).

### Data analysis

Our analytic sample included the CHWs (*n* = 5) and participants who completed baseline surveys at the time of their baseline assessment and had full responses to mental health symptoms (*n* = 37). We excluded 6 enrolled participants in the post-intervention analysis for Organization B—2 were lost to follow-up, and 4 did not attend any ALMA sessions after enrollment. We used descriptive statistics to assess the frequencies, percentages, means and standard deviations for all variables. We assessed whether the CHW facilitators completed all components of the curriculum for each session using data from the weekly fidelity survey and CHW facilitator support meeting notes. We also reviewed our weekly CHW facilitator support meetings notes and open-ended responses to the weekly fidelity survey for themes regarding curriculum implementation to identify future recommendations. We captured themes related to what went well during sessions, what did not go well, rationale for missed components of intervention delivery, and modifications to activities.

## Results

### Implementation outcomes

All CHWs that were trained as ALMA facilitators were Latina and Spanish-speaking with several years of experience serving Latina immigrants through their CBOs. CHWs also had previous experience facilitating educational programs for community members. The mean age of the facilitators was 45 years. All were born in Mexico and had at least a high school diploma or equivalent. The facilitators had lived in the US for a mean of 26 years. Four out of five of the facilitators had worked in their CBO for <5 years. All CHWs attended all parts of the training.

Facilitators’ ability to complete all curriculum components varied. Organization A canceled the last session during their delivery of the ALMA intervention due facilitator availiability. Components from this session were combined with the previous session. Organization A completed all activities except one in the second session (see Table 1). Organization B completed all activites except two in session 1, one activity in session 4 and one activity in session 5 (see Table 1). Reasons for missing activities included running out of time or facilitators forgetting to include a component of the curriculum. Facilitators reported difficulty keeping track of time, especially when they wanted to ensure all participants had a chance to share. Similarly, facilitators noted that when attendance was lower there was more time for each participant to share. Some facilitators missed sessions due to illness or medical appointments, which required other facilitators to cover their planned content. There was no variation in session length at either organization. Some facilitators reported making small modifications to activities, such as completing an activity in the larger group instead of in multiple smaller groups or switching the order of activities to better fit their physical space constraints. Finally, facilitators requested additional training on what to do if a participant expressed serious mental health symptoms, like suicidal ideation.

**Table 1:** ALMA sessions and program activities.

Session topic	Content
1. Arriving, connecting, introductions	Welcome, introduction to the program, and group introductions Discussion of mental health, stress, and coping Group Norms: “Acuerdos”** Discussing coping strategies: “herramientas” Body awareness practice (Relaxation) ALMA body drawing** Reflection
2. Telling our stories of migration	Breath awareness practice Song: “Inhalando, exhalando” Story circles: Sharing our stories of migration Belly button energizer Mindful movement: working with emotions in the body Body awareness practice (Relaxation)* Reflection
3. Stress and life here in central Washington	Song: “Inhalando, exhalando” Breath awareness practice ‘The Tree of Stressors’: stress and immigration-related stressors Sponge activity: Stress metaphor Mindful movement: working with stress in the body Body awareness practice (Relaxation) Reflection
4. Interconnectedness and support: coming home to ourselves and each other	Song: “Inhalando, exhalando” Spider web activity: Identifying emotions and sources of interconnection and support Mindful eating: connection with/through food Body awareness/self-compassion practice Reflection Emotion drawings: discussing difficult emotions Mental health resource handout**
5. Working with challenging emotions	Song: “Inhalando, exhalando” Affirmations for one another Body awareness and relaxation: self-compassion in difficult times Movement: Moving through difficult emotions Self-compassion letters**
6. ALMA in our daily lives and end of program celebration	Potluck style dinner Dichos: Reflecting on words of wisdom from loved ones Song: “Inhalando, exhalando” Breathe awareness practice Mindful movement Body awareness and relaxation Gratitude circle and celebration

*Indicates activity that was missed by Organization A. **Indicates activity that was missed by Organization B.

### Participant outcomes

**Figure 1:**
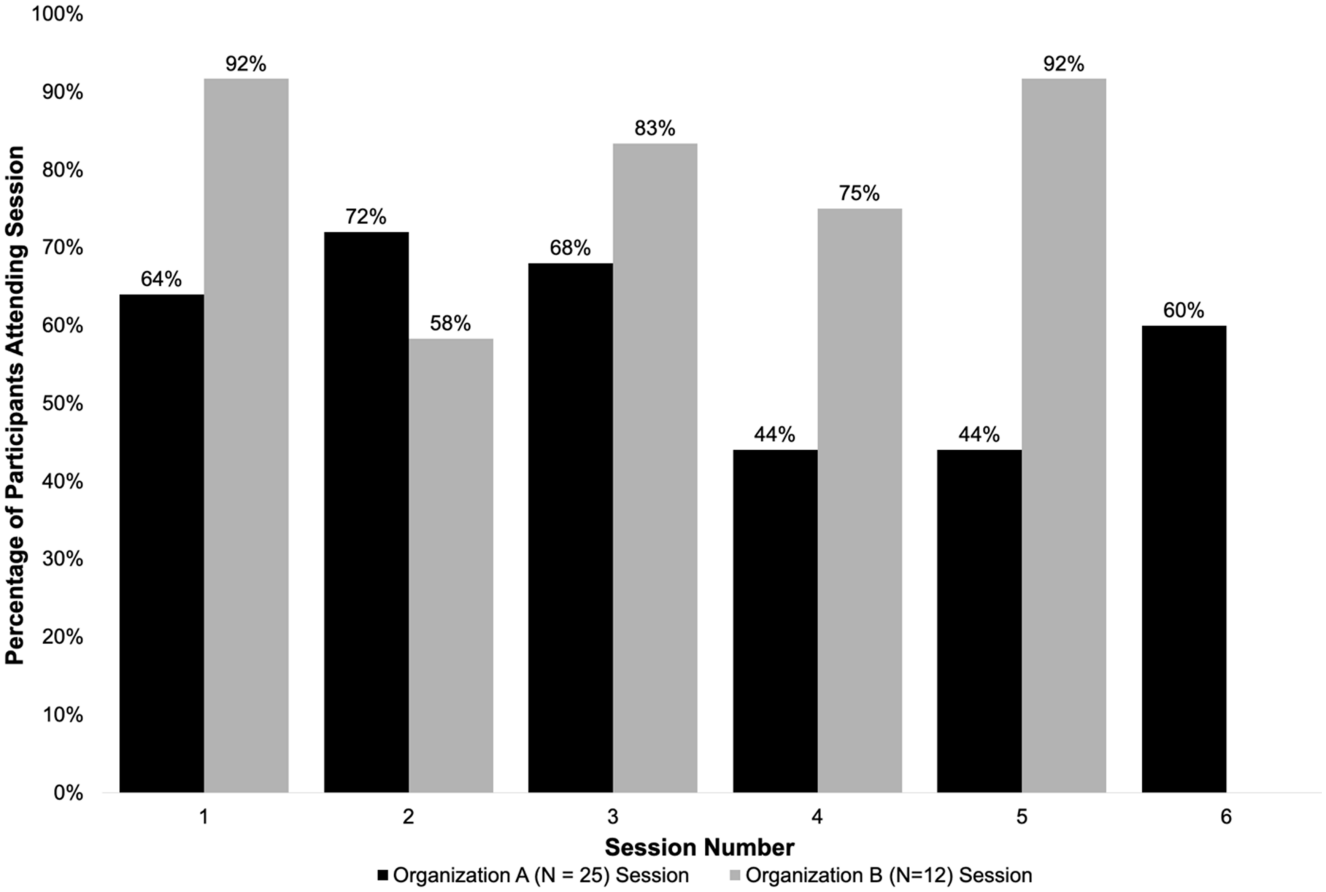
Attendance of enrolled participants.

The sample had a mean age of 45 years, most participants were from Mexico (89%) and had lived in the US for an average of 16 years. Most participants had less than a high school degree or equivalent (73%), were not currently working (78%) and were low income. Overall, participants reported mild levels of depression and anxiety symptoms at baseline. Participant attendance for each session of the CHW-facilitated intervention is shown in Figure 1. On average, participants from both sites attended 70% of the sessions. For Organization A, the average attendance rate for the sessions that were held was 80% (*N* = 12). Organization B, which delivered all six sessions to their group, had an average attendance rate of 59% (*N* = 25).

Participants reported high levels of satisfaction with the CHW-facilitated ALMA intervention overall (see Table 2). On average, participants reported using the skills they learned in ALMA several times a week (see Table 2). Changes in depression and anxiety scores from pre-to post-intervention are shown in Table 3. Overall, mean depression scores (PHQ-8) decreased from 7.2 (mild severity) pre-intervention to 4.5 (minimal severity) post-intervention, and anxiety scores (GAD-7) decreased from 6.7 (mild severity) to 3.8 (minimal severity).

**Table 2:** Participant satisfaction and skills learned (*N* = 31).

	Overall (*N* = 31)		Organization A (*N* = 12)		Organization B (*N* = 19)	
	M	SD	M	SD	M	SD
Participant satisfaction						
*Program satisfaction*						
I would recommend this program to my friends, family, or other women	4.7	0.5	4.8	0.4	4.6	0.5
I think the information provided was relevant for women like me	4.7	0.5	4.7	0.5	4.7	0.5
I liked the ALMA program	4.7	0.4	4.8	0.5	4.7	0.5
*Program logistics*						
The time and duration of the sessions was convenient so I could participate	4.5	0.7	4.3	1.0	4.6	0.5
*Perceived efficacy*						
ALMA helped me manage stress	4.5	0.6	4.6	0.5	4.5	0.6
ALMA helped me recognize difficult emotions	4.5	0.6	4.6	0.5	4.4	0.6
ALMA helped me feel self-compassion	4.6	0.5	4.6	0.5	4.6	0.5
ALMA helped me feel less alone	4.5	0.5	4.5	0.5	4.6	0.5
ALMA helped me relax	4.7	0.5	4.7	0.5	4.7	0.5
ALMA helped me learn how to relax	4.6	0.6	4.8	0.5	4.5	0.6
ALMA helped me find new strategies to reduce the stress in my life	4.5	0.6	4.8	0.5	4.4	0.6
The information that I received in the program helped me improve my mental health	4.5	0.6	4.6	0.5	4.4	0.6
Skills learned						
Pay attention to my breath	3.2	0.7	3.3	0.5	3.1	0.8
Paying attention to what I am feeling in my body	3.4	0.8	3.7	0.5	3.2	0.9
Paying attention to stressful moments and difficult emotions	3.0	0.9	3.4	0.5	2.7	1.1
Paying attention to what I eat	3.2	1.2	3.3	0.7	3.1	1.5
Feel connection (with loved ones and with the earth)	3.2	0.9	2.9	0.9	3.3	0.9
Move my body to release tension	2.9	1.2	3.3	0.7	2.6	1.5
Be affectionate with myself and take care of myself	3.1	1.1	3.3	0.6	3.0	1.3
Be grateful to myself and to others for the things I/they do	3.3	0.9	3.4	0.5	3.3	1.1
Relax my body	3.4	0.9	3.3	0.7	3.4	1.1

**Table 3:** Pre-and post-intervention depression and anxiety scores.

	Depression			Anxiety		
	Overall (*N* = 37)	Site A (*N* = 12)	Site B (*N* = 25)	Overall (*N* = 37)	Site A (*N* = 12)	Site B (*N* = 25)
Pre-intervention Mean (SD)	7.2 (5.0)	7.6 (3.8)	7.0 (5.6)	6.7 (5.2)	6.5 (3.7)	6.8 (5.9)
Post-intervention Mean (SD)	4.5 (4.8)	3.6 (3.1)	5.0 (5.5)	3.8 (4.9)	2.3 (2.7)	4.6 (5.6)

## Discussion

This pilot study assessed the feasibility of delivering the ALMA intervention with CHWs in a setting with a limited culturally and linguistically appropriate mental health workforce. CHWs delivered most components of the intervention and participants had good attendance and reported high satisfaction with the program. Those that participated in the intervention had reductions in their depression and anxiety symptoms. Our findings suggest the need for further research on how to best train and support community health workers delivering evidence-based mental health interventions.

Previous studies have assessed the feasibility of CHW-delivered mental health interventions in the US, including at least one study with Latino participants, but very few have assessed this approach for interventions targeting Latina immigrants (17). Our findings showed that when CHWs receive training and ongoing support during facilitation they can deliver mental health interventions. We also found that it may be necessary to enhance CHW training to build more familiarity with the curriculum and reduce missed components. Training and support should also enhance or support CHW delivery in a way that reflects their unique strengths, insight and familiarity with the community. Our training was comparable to previous studies that have reported a wide range of CHW training lengths (2 days to 3 months) (18). Some participants also reported wanting either longer sessions or more sessions in order to have more time to share. Limiting the group size could make facilitation easier and help ensure there is enough time for each activity.

The characteristics of the participants and their outcomes were similar to those enrolled in the ALMA intervention trial which was delivered by mental health and mindfulness professionals (12). Participant satisfaction was high overall and similar across organizations. Depression and anxiety scores also decreased. Scores for all outcomes were also consistent with the intervention trial, which indicates that this implementation strategy has the potential to have comparable levels of effectiveness to when it was delivered by professionals (12, 13). These findings suggest that this implementation strategy was acceptable and has the potential for a high impact.

CHWs offer a promising approach to delivering the ALMA intervention to Latina immigrants in settings where there are few Spanish-speaking mental health providers and mindfulness experts. Results indicate that CHWs may need enhanced training and support to ensure high levels of fidelity. Our findings were limited to a small sample in a specific region of Washington state, which may limit the generalizability of our findings. Our implementation outcomes were self-reported by the CHWs, which may have introduced bias. Future studies should include more rigorous approaches to assessing fidelity, such as recordings or observers, as well as larger samples with more rigorous study designs.

## Data Availability

The raw data supporting the conclusions of this article will be made available by the authors, without undue reservation.
